# The Role of Incomplete Information and Others' Choice in Reducing Traffic: A Pilot Study

**DOI:** 10.3389/fpsyg.2016.00135

**Published:** 2016-02-11

**Authors:** Angelo Romano, Cristina O. Mosso, Ugo Merlone

**Affiliations:** ^1^Psychology Department, University of TurinTurin, Italy; ^2^Center for Cognitive Science, University of Turin and Polytechnic of TurinTurin, Italy

**Keywords:** Braess paradox, cooperation, traffic networks, social processes, social comparison, uncertainty, Social dilemmas

## Abstract

In this study, we investigate the role of payoff information and conformity in improving network performance in a traffic dilemma known as the Braess paradox. Our goal is to understand when decisions are guided by selfish motivations or otherwise by social ones. For this purpose, we consider the manipulation of others' choice, public and private monitoring and information on distribution of choices. Data show that when social comparison was not salient, participants were more cooperative. By contrast, cooperativeness of others' choice made participants more competitive leading to traffic and collective performance decrease. The implications of these findings to the literature on social dilemmas are discussed.

## 1. Introduction

The operation of transportation and communication in modern societies relies on networks (Rapoport et al., 2009). For this reason, the management and the improvement of such networks are becoming essential problems in modern society. An intrinsic characteristic of these networks is that they are the social spaces in which individuals act selfishly. Therefore, it is important to investigate the emergence of some detrimental properties derived by individual behavioral patterns. When considering networks, according to Rapoport et al. (2006) one of the main questions to be addressed is how behavior adapts to changes in the network structure in decentralized systems. Indeed, answers to this question could be interesting for both managers and scientists.

In this paper, we present a pilot study aimed to improve the network performance in the Braess paradox (Braess, 1968). The Braess paradox is defined as the counter-intuitive phenomenon in which the introduction of a route in a traffic network leads to a traffic increase rather than a decrease (Braess, 1968). We conducted a pilot study to increase our understanding of whether a social psychological perspective can help to reduce traffic in the Braess paradox. We focused our attention on structural solutions for social dilemmas and found support for the hypothesis that incomplete information may reduce traffic.

The paper is divided in 7 sections. In Section 2, we provide a definition of the Braess paradox and present some instances of real life examples. In Section 3, we argue that the Braess paradox—under a social psychology perspective—can be considered as a social dilemma and present some variables that have been considered to tackle collective disasters in these situations. In Section 4, we present the rationale and design of our study. In Sections 5 and 6, the methods and the results of the experiments are presented. In Section 7, we discuss the results and provide some directions for future research.

## 2. The braess paradox

**Figure 1:**
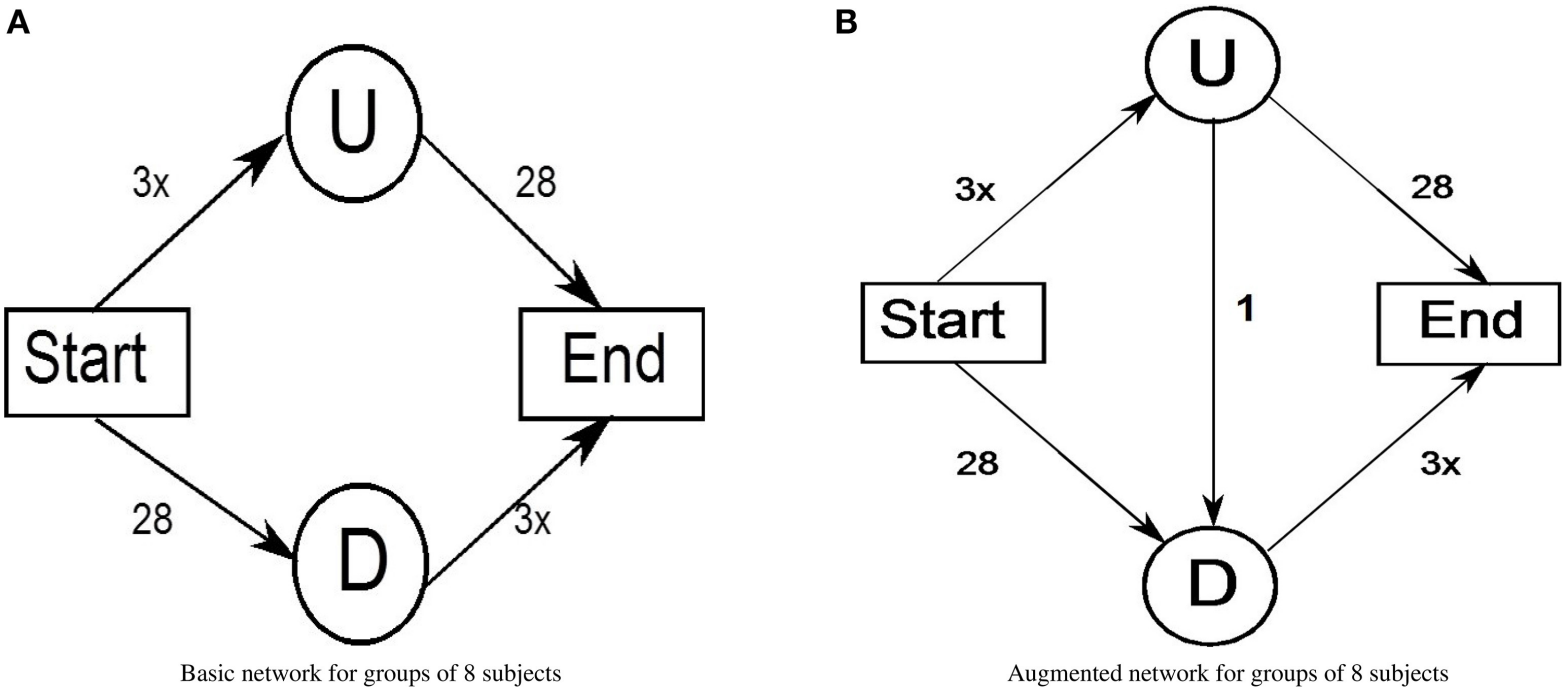
**Example of Braess paradox traffic network**. **(A)** Basic network for groups of 8 subjects. **(B)** Augmented network for groups of 8 subjects.

Consider a population of 8 individuals playing several rounds in two phases: in the first phase subjects play in the so-called *basic network phase* (see Figure 1A). They are asked to imagine that they are commuters that every day (round) have to go from the node Start to the node End in the shortest time possible. As commuters, they can decide between two possible routes, the first one Up consists of passing through the node U and the second one Down consists of passing through the node D. Both routes consist of two links, one with fixed travel time (Start-D and U-End), and one depending on congestion (Start-U and D-End). As it concerns the first link, travel time is 28 min, and for the second link, travel time increases linearly with the number of commuters. In this situation, rational commuters will equally distribute between the two routes. Thus, the expected traffic (travel cost) for the commuters in this situation will be about 40 min. In the second phase of the game, participants play the same type of game but with the introduction of a third possible route derived by a link with a constant travel time of 1 min between the node U and the node D. This phase is called the *augmented network phase* (Figure 1B). Specifically, the new route is made by the nodes Start-U-D-End: we will call this route UD. In this case, we have a Nash equilibrium, i.e., a choice configuration where each player's choice is optimal given the choices of all other players and so all rational commuters will converge into the new route UD. With this new equilibrium, all participants will spend about 49 min to reach the point End, with the paradoxical result of increasing both congestion and travel time as it occures also in other examples with different travel times (Rapoport et al., 2009).

The Braess paradox has been object of interest for several disciplines such as transportation science (Steinberg and Zangwill, 1983), and computer science (Roughgarden, 2006). Previous research on the Braess paradox reveals that it is a common event rather than a rare one. For example, when considering a large class of random networks, the probability of the paradox occurring is very high (Steinberg and Zangwill, 1983; Roughgarden, 2006). According to Gisches and Rapoport (2012), in the last 40 years the Braess paradox was considered a theoretical issue by many researchers. However, we can find many empirical examples of the Braess paradox. Murchland (1970) noticed that in the city of Stuttgart there were a series of failed attempts to improve the traffic when adding new routes. Youn et al. (2008) analyzed more than 200 roads in Boston and found several examples of the Braess paradox. Similar findings were observed by Kolata (1990) and Fisk and Pallottino (1981) in the city of Winnipeg. Empirical examples of Braess paradox can be found also in electricity (Pala et al., 2012), and team strategy (Skinner, 2010).

## 3. Social psychology and the braess paradox: A social dilemmas perspective

According to Arnott and Small (1994), the Braess paradox can be considered a type of social dilemma due to the negative externalities derived by following selfish strategies. Considering the Braess paradox as a social dilemma allows for the investigation of social psychological factors to reduce the damaging effects of structural changes in traffic networks. Social dilemmas are pervasive and present in several aspects of our daily life. For this reason, the study of cooperation in social dilemmas is one of the most interesting issues for psychologists (Dawes, 1980), economists (Ostrom, 1998), and sociologists (Kollock, 1998). Communication, group-identity and self-efficacy are just few examples of the variables considered to avoid the collective disaster of social dilemmas (Liebrand, 1984; Brewer and Kramer, 1986; Komorita and Parks, 1996; De Cremer and Van Vugt, 1999). Research has also focused on factors such as reputation (Wu et al., 2015), trust (Irwin et al., 2015), and punishment (Xiao and Kunreuther, 2015).

Social dilemmas are mixed-motive interdependent situations in which there is tension between individual rationality and collective welfare and can be classified in commons dilemmas and public goods dilemmas (Dawes, 1980). In both situations, the outcome for an individual depends on the decisions of all parties involved. Commons dilemmas are situations where individuals have the temptation to take more than their part of a common source in the short-term, but if all individuals behave selfishly this will lead to a collapse of the common good in the long-term (Van Lange et al., 2013). In public goods dilemmas the selfish temptation is to avoid the the short-term cost of contributing to a collective good, which leads to the common source not being provided in the long-term. According to Dawes (1980), social dilemmas are characterized by two necessary properties. The first is the presence of a dominant strategy, while the second is the presence of a deficient equilibrium. The presence of dominant strategy refers to situations in which for every individual the payoff associated with the defective choice is higher than the cooperative one, independently from what the others decide (Osborne and Rubinstein, 1994). Since the outcome related to the dominating equilibrium is less than the cooperative one, this outcome is called deficient equilibrium. In other words, ‘in social dilemmas each decision maker is best off acting in his/her own self-interest, regardless of what others do; yet, each self-interested decision creates a negative outcome for all involved’ (Wade-Benzoni et al., 1996, p. 111).

In the Braess paradox, the route derived by the introduction of the new link, UD, in the *augmented network phase* is the dominant strategy, and the equilibrium reached is Pareto dominated by the equilibrium of the *basic network phase*, that is the one where individuals split equally between the route Down and the route Up. For this reason, rather than being a simultaneous game as is the prisoner's dilemma, i.e., a game where cooperative or competitive dynamics depends on the decisions made by individuals at the same time, according to Rapoport et al. (2006) the Braess paradox can be considered a structural dilemma because the negative effects of individual rationality depend on structural network changes. Furthermore, the augmented route is formed by the two links that depend on congestion (Start-U and D-End). These links are also part of the Up route (Start-U), and the Down route (D-End). Therefore, a person who chooses the augmented route increases the traffic of all commuters in the network. Conversely, choosing one of the other routes increases the traffic only in that route. For this reason, each participant who avoids the augmented route is always helpful to the collective.

In this context, in the Braess paradox individuals have the possibility to experience a better outcome in the *basic network phase*. Nevertheless, once introduced to the new route, congestion and also individuals' travel times increase. Therefore, in the Braess paradox cooperation consists of avoiding the choice of the new route UD once it has been introduced. This is coherent with the definition of cooperation in social dilemmas: the behavior of an individual who acts against immediate individual interest, but one that is beneficial to all if a sufficient fraction, or the totality, of individuals adopt it (Boyer and Orléan, 1997).

## 4. The rationale of the study

The Braess paradox has been revealed to be robust in lab experiments even when varying the number of commuters, roads, number of turns played by participants, and also when reversing the two phases of the game (Rapoport et al., 2009; Gisches and Rapoport, 2012). These evidences suggest that reducing this phenomenon may prove to be difficult. In fact, according to Rapoport et al. (2006), in the Braess paradox variables as tacit coordination, altruism, reciprocity and punishment are less effective.

According to the interdependence theory (Van Lange and Rusbult, 2011; Van Lange et al., 2013), in mixed motive interdependent situations, choices depend on the interaction of structural factors (e.g., the type of social dilemma, social situation), psychological influences (motives, affects cognitive aspects), and interaction dynamics (e.g., the strategies of other partners).

In this study we present the Braess paradox as a paradigm to test some factors used in social psychology literature. In particular, we decided to manipulate factors that are recognized to be structural solutions to social dilemmas (Kollock, 1998): others' choice, payoff information and distribution information. In fact, these structural interventions are particularly suitable to investigate some social processes.

Table 1 shows the design of the experiment. Below our main hypotheses according to the design are briefly summarized.

**Table 1:** **The 8 conditions of the 2 × 2 × 2 design of the experiment**.

	**Public distribution**		**Private distribution**	
	**Cooperative Population**	**Non-cooperative population**	**Cooperative population**	**Non-cooperative population**
Public payoff	1	2	3	4
Private payoff	5	6	7	8

### 4.1. Others'choice

As discussed in Section 3, in the *augmented network phase*, following the dominating equilibrium, i.e., the new route introduced, increases traffic. We discussed that the previous equilibria, i.e., the ones in the *basic network phase*, can be compared to the cooperative strategy that has to be promoted in other social dilemmas. We decided to investigate whether playing with a cooperative population may reduce traffic. Accordingly, a cooperative population is defined as a set of preprogrammed individuals' choices that—once introduced the route UD-maintain the equilibria of the *basic network phase*.

Studies investigating the role of others' choices in social dilemmas show that a cooperative environment can likely be exploited by individuals (Oskamp, 1971; Wilson, 1971; Patchen, 1987). However, there are cases in which conformity factors enhance cooperation (Samuelson et al., 1984). We expect that interacting with a cooperative population will increase cooperation. In a recent study conducted by Parks et al. (2015), two types of information were manipulated in order to investigate behavior in a commons dilemma. The Authors considered *environmental information*, the information about the actual state of the resource, and *social information*, the information about what others choose and earn in each round of the game. We focus on the *social information* factor by splitting it in two components: one related on the simple information about what others choose, i.e., distribution information, and one related on the payoff that others obtain with their choices, i.e., payoff information.

### 4.2. Distribution information

Others' choice can be made salient by showing the information on how the number of people opting for one route differ from one another. As in a recent experiment on information in resource dilemmas conducted by Parks et al. (2015), we want to investigate whether knowing what others decided in the past can influence individual behavior regardless of the personal interest. Conformism has been revealed as a strong factor influencing decision-making (Asch, 1951; Samuelson et al., 1984; Bardsley and Sausgruber, 2005; Szolnoki and Perc, 2015). Moreover, findings from social psychology (Joireman et al., 1997; Irwin and Simpson, 2013; Van Lange et al., 2013) have widely demonstrated that peers have a huge influence on individual decisions. In our design, the highest activation of conformism processes is expected when interacting with a cooperative population and when the information about the distribution of others' choices is salient. The distribution information regards only the number of subjects choosing one route over another. Therefore, according to this social process, we expect that individuals will conform with the choices of others and behave more cooperatively. We will then compare these two hypotheses.

### 4.3. Payoff information

If the distribution information makes individuals aware of what the others decided in the past, payoff information makes the outcomes obtained by each participants salient in every round. As in the Braess paradox experiment participants accumulate points by reducing the time taken to reach the point End, this information can make clear how social (i.e., the number of individuals related to a choice) and rational selfish calculations (i.e., the payoff associated to each choice) can interact together in this situation. According to Parks et al. (2015) the information about others' payoff increases concrete thinking about how well participants are doing in comparison to others, and this reasoning may decrease cooperation. The possibility of a comparison between the personal payoff and others' could increase competitive behavior (McClintock and McNeel, 1966; McNeel, 1973). Subjects have been given different kinds of information about the payoff obtained in each trial: one group of subjects was informed of others' payoff whereas a different group of subjects was informed of just their own payoff. In our point of view, the manipulation of payoff information makes the role of social comparison salient. Gisches and Rapoport (2012) found no differences between private and public monitoring; their study differs from ours in terms of the number of subjects (18) and trials (120). The number of trials and group size have been revealed an important factor enforcing the Braess paradox (Gisches and Rapoport, 2012). Regarding the activation of social comparison processes our aim is to understand whether comparison triggers selfish choices. Research on cooperation in social dilemmas has shown that individuals have particular concerns for fairness (Van Lange, 1999; Stouten et al., 2005, 2009; Murphy et al., 2011). Accordingly, we expect more competition in the public payoff condition in comparison to the private payoff one: this is our third hypothesis.

In summation, the manipulation of others' choice allows us to investigate the effect of interacting with different kinds of players (cooperative vs. non-cooperative populations), payoff information and distribution information have the ability to make the role of social comparison and conformity salient.

To the best of our knowledge, the are no studies in which the Braess paradox has been significantly reduced. In our opinion, this study contributes to the social dilemmas literature by:

- investigating the role of social processes in improving collective performance in the Braess paradox;
- proposing a new paradigm to promote cooperation in social dilemmas.

## 5. Materials and methods

### 5.1. Participants

Our sample size depended on the guideline provided by Isaac and Michael (1971) to have 30 participants for a pilot study, and also on the suggestion to have around 10% of the total sample size (Treece and Treece Jr, 1977). To extend this study, a power analysis (Faul et al., 2007) suggests to have about 220 participants per cell in order to detect a power of 0.08 with a medium effect size and an α of 0.01.

A total of 151 Psychology and 109 Business administration students (100 male and 160 female) from the University of Turin, all aged between 18 and 21 years, were recruited in a 2 × 2 × 2 between-subjects experiment. Participants were randomly assigned to the 8 conditions.

### 5.2. Materials and procedure

This study was carried out in accordance with the recommendations of “Deontological Code of Italian Psychologist” (see art.9) with written informed consent from all subjects. We received written informed consent from all subjects in accordance with the Declaration of Helsinki. Regarding the ethics of our study we replicated the experimental design already validated in the literature (Rapoport et al., 2009) in which no ethical approval was requested. In fact, the participants were asked to choose a route in a traffic situation without any implicit inferences or psychological indirect measure. The data were examined collectively in accordance with the declaration that was written on the consensus that participants signed in order to understand the aim of the study and the procedure of our data analysis.

In this study, we ran our experiment using the computerized experimental lab of the University of Turin with the software z-Tree (Fischbacher, 2007). Data have been collected in a lab within different computers in order to simulate a real interdependent session of the game.

Participants were seated in different cubicles and then they read the instructions of the *basic network phase* (see Appendix A). After the instructions, they were asked to answer some comprehension questions followed by the first 8 rounds. After each round, participants received feedback on their travel time in reaching the point End. All feedback was different according to the different manipulations (see the examples of different manipulations in Appendix C from Tables C1–C4). In the *public payoff condition* and *public distribution condition* participants were aware of the travel times and distribution of choices of all the other participants in their session, while in the *private payoff condition* and *private distribution condition* they only read their own payoff. The same procedure was followed for the *augmented network phase* (see Appendix B). Each session was conducted with groups of 8 subjects who were led to believe they were playing together, while, actually their feedback was predetermined according to the different *others' choice* conditions (see Appendix C in Tables C5–C7).

With the aim to trigger motivation, participants were informed that the more points they earned the higher was their probability to win 1 kg of chocolate in a lottery at the end of the session. A lower time in reaching the point End, meant more the points accumulated by individuals in each round (see Appendix A for the formula on the accumulation of points). Although economists and psychologists do not usually agree that incentives improve performance in laboratory experiments (Gneezy and Rustichini, 2000), according to Smith (1991) there is enough evidence that incentives increase motivation. Subjects played 8 rounds in the first phase and 8 rounds in the second one.

Below we summarize the operationalization of the three independent variables: Others' choice (cooperative vs. non-cooperative), Payoff information (public payoff vs. private payoff) and Distribution information (public distribution vs. no-distribution).

- *Others' choice*. In our study participants were led to believe that they were playing with a real group while, actually, they played against fixed patterns of choices by other participants. A group of subjects played with a cooperative population whereas another group of subjects played with a non-cooperative one. In the *basic network phase*, all subjects played with the same kind of fictitious population with choices determined by previous experiments. In the *augmented network phase*, each participant could interact with two different types of artificial populations: a cooperative and a non-cooperative population. According to the definition of cooperation provided in Section 3 with cooperative population we refer to a situation in which nobody chooses the new route, while the non-cooperative population condition choices were based on a pre-test conducted with 37 participants playing a real session of the Braess paradox, and on other real experiments with human participants provided in literature (Dal Forno and Merlone, 2013). To be more specific, this has been computed as the mean of choices made for each turn in the experiments conducted with real sessions of the Braess paradox. For this reason, the non-cooperative population can be considered as a control condition.
- *Payoff information*. In the public-payoff condition participants were informed at each turn what they and others obtained. In the private-payoff condition, participants just observed their own payoff.
- *Distribution information*. In the distribution information condition, participants had the possibility to see how decisions are distributed among participants. To be more specific, subjects in the public distribution information condition were informed about the choices distribution of the other fictitious participants in their group (e.g., 5 subjects chose Up and 3 subjects chose the route Down), whereas the other group was not informed of the distribution information.

The dependent variable is cooperation. Cooperation is defined as a lack of exploitation of the new route UD during the 8 rounds of *augmented network phase*. More specifically, it has been measured considering the number of times in which subjects chose to exploit the route UD. Therefore, cooperation is associated with small values, and a small mean indicates a cooperative tendency.

## 6. Results

We conducted a 3-way ANOVA to test our hypothesis. Table 2 shows the mean and standard deviation for each condition. As we can observe in Table 3, we have a main effect of payoff information condition, an almost significant effect of others' choice and three significant interaction effects. Considering the payoff information, playing without the possibility of comparing the proper travel times obtained with the others seems to decrease the exploitation of the route, *F*_(1, 252)_ = 7.988 *p* = 0.005.

**Table 2:** **Descriptive statistics of the rate of route UD choices in the 8 conditions**.

**Others' choice**	**Payoff information**	**Distribution information**	***N***	**Mean**	**Standard Deviation**	
Cooperative	Private	Yes	39	5.333	2.286
		No	30	5.600	2.127
	Public	Yes	29	6.206	1.952
		No	32	4.718	2.358
Non-Cooperative	Private	Yes	39	3.538	1.903
		No	30	4.866	1.306
	Public	Yes	29	5.448	1.938
		No	32	6.000	2.918

**Table 3:** **Summary of the main results of a 3-way ANOVA**.

	***F***	***p***	**$\eta_{p}^{2}$**
Others' choice	3.487	0.060	0.014
Payoff information	7.998	0.005^*^	0.031
Distribution information	0.376	0.540	0.001
Distribution information × Others' choice	8.339	0.004^*^	0.032
Distribution information × Payoff information	5.555	0.019^*^	0.022
Payoff information × Others' choice	8.069	0.005^*^	0.031
Distribution information × Payoff information × Others'choice	0.830	0.363	0.003

*Significant difference considering α = 0.01*.

### 6.1. Interactions

**Figure 2:**
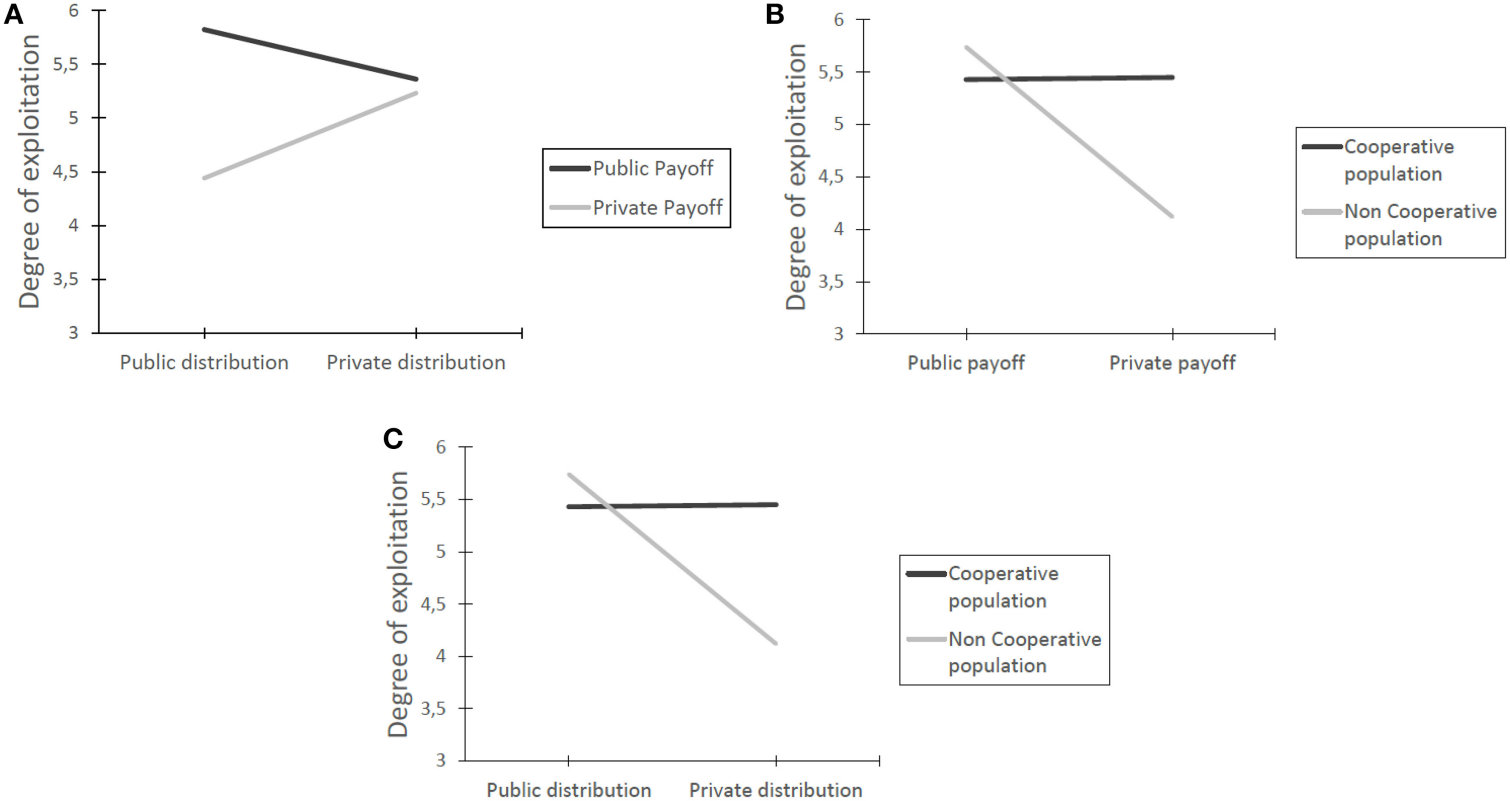
**Interactions among the experimental conditions**. **(A)** Payoff and distribution information interaction. **(B)** Others' choice and payoff information interaction. **(C)** Others' choice and distribution information interaction.

The payoff and distribution information interaction presented in Figure 2A, shows that when payoff is public, the information about others choices undermines cooperation. Conversely, when payoff information is private, the distribution of information increases cooperation: this is coherent with the idea that once individuals have no information on their outcomes, they rely on the behavior of others (Molleman et al., 2014; Parks et al., 2015). Looking at the cooperativeness of others' choice and payoff information (Figure 2B), the positive effect on cooperation of the private payoff condition can be observed when interacting with a non-cooperative population but seems to have no effect with a cooperative one. This can be due to the fact that when people select the UD choice in the cooperative population, the favourable outcome related to this choice does not provide reasons for people to change their route. Finally, regarding the distribution of choice in comparison with the cooperativeness of a population (Figure 2C), public distribution information enhances the exploitation of the UD route. This can be due to the fact that the cooperative population, see Table C6, makes public that no one choose the new route and therefore this provides more opportunities to earn points by behaving selfishly. Accordingly, this explanation can be used to explain the opposite trend when interacting with a non-cooperative population. As in this situation the majority of individuals chooses the route UD, individuals try to earn more points choosing the other routes, i.e., Down and Up.

## 7. Discussion

In our study, we presented a traffic network based on a commons dilemma known as the Braess paradox and tried to reduce the exploitative behavior by making some crucial social processes salient. The most relevant finding of our investigation was the effect of private monitoring in minimizing the use of the newly introduced route: incomplete information about payoff seemed to be effective in reducing selfish behavior in the Braess paradox. Therefore, information on others' behavior can undermine cooperation in traffic networks dilemmas. This result replicates the findings found by Parks et al. (2015) in a resource dilemma. According to Stouten et al. (2009), a possible explanation for this effect is that when individuals are aware of asymmetric distribution of resources, they tend to apply fairness rules to fill the gap (distributive justice). Moreover, the presence of some individuals regretting unfair distribution of resources is suggested by recent research on Social Value Orientation (SVO) (Murphy et al., 2011). In fact, according to Murphy et al. (2011), inequality aversion leads individuals to reach fairness, even if this fairness decreases the collective welfare. Our results are also coherent with the model of inequality aversion provided by Fehr and Schmidt (1999). According to the authors, people may exhibit a preference for equal distributions. They argue that agents with fairness considerations tend to punish those who are perceived as the cause of an unequal distribution of resources. Therefore, according to this model, in the public payoff condition a participant may choose the augmented route to reduce the difference between his/her outcome and the one of the other participants.

Following the suggestion given in Parks et al. (2015), we addressed the role of cooperativeness of others' choice in this incomplete information scenario. In this study we address this point and our results suggest that interacting with a cooperative population seems to enhance competitive decisions rather than cooperative ones. This result provides more support for the rational individual perspective suggesting that in situations in which individuals can obtain the maximum payoff, social processes such as conformity do not work. The distribution information seems to have no relevance on cooperative choices in the Braess paradox. According to Irwin and Simpson (2013), others' choice also have an important influence in cases where it is clear that the majority choice is wrong. Following these lines, we believed that distribution information could play a role in the cooperative population condition because in such a fictitious population no one was programmed to use the route UD. This kind of findings provide robustness of the rationality explanation in social dilemmas and for the phenomenon of Braess paradox. Accordingly, these results provide support to the literature on evolutionary game theory suggesting that when we introduce defectors in a cooperative population, the latter population will likely be exploited (see Ohtsuki et al., 2006; Perc and Szolnoki, 2010; Wang et al., 2015).

Finally, it seems that individuals can be guided by both rational and social factors according to the condition they are immersed in Kelley and Thibaut (1978). This is an interesting insight because individuals are not seen as exclusively rational or situation-dependent but rather as individuals acting differently according the situation and social context in which they are immersed. As this represents a pilot study, an important limit represents the power of the significant results and interpretation of null findings. It is possible that with increasing the sample size the effect of payoff information may diminish, showing for example that social influence has a minor role in the Braess Paradox.

Future research on social dilemmas may focus on other factors involved in decisions, taking into account both structural and psychological aspects. From our point of view, following these lines could be an effective strategy to promote cooperation in social dilemmas, and a possible way to shed light on the processes involved in decision-making. To be more specific, the findings of our study are related to situations in which the number of both subjects and turns is limited. In further research, it may be noteworthy to focus on generalizing these results to different group-sizes and to a larger number of turns in order to understand the extent of the social factors examined. Another possibility for advancement could be the analysis of the role of other psychological factors as group-identity in minimizing selfish decisions or exploring more deeply the fairness explanation by introducing punishment or by investigating individual differences in terms of social value orientations.

## Author contributions

All authors listed, have made substantial, direct and intellectual contribution to the work, and approved it for publication.

### Conflict of interest statement

The authors declare that the research was conducted in the absence of any commercial or financial relationships that could be construed as a potential conflict of interest.

## Appendix

### A. Instructions basic network phase

- Imagine you are a commuter who must travel every day from Start to End. You can choose two possible routes: one is the Up road and the other one is the Down road.
- Your goal is to reach End in the shortest possible time.
- Down road and Up road consist both of two segments: for the first one travel time is always 28 min, and for the other one it depends on the traffic congestion in the route.
- Let P be the number of commuters who will be on the route at the same time.
  If you choose the Up route, the section from Start to U has a travel cost of 24/P min times every commuters in that section; the section from U to End has a constant travel time of 28 min.
- If you choose the Down route, the section from Start to D takes a constant travel cost of 28 min and the section from D to End takes a travel time of 24/P multiplied by every commuter in that section.
- You will accumulate points depending on how much time you have saved: $\frac{Your\;travel\;time-50}{2}$.
- You cannot communicate with other participants

### B. Instructions augmented network phase

- A bridge with a constant travel time of 1 min has been built from U to D. You have now another way to reach End from Start. The new choice UD consists of three parts, the first segment of the route Start to U,U to D, and the second segment of D to End. The travel costs of other choices, Up and Down, remain the same as in the Basic Phase.
- You goal is to reach End in the shortest possible time.

### C. Example of different conditions and artificial population

**Table C1:** **Feedback example of *public payoff condition* and *public distribution condition***.

**Your decision**	**Down**
Travel time of participants who chose Up	37
Travel time of participants who chose Down	43
Number of Players choosing Up	3
Number of Players choosing Down	5

**Table C2:** **Feedback example of *public payoff condition* and *private distribution condition***.

**Your decision**	**Down**
Travel time of participants who chose Up	37
Travel time of participants who chose Down	43

**Table C3:** **Feedback example of *private payoff condition* and *public distribution condition***.

**Your decision**	**Down**
Your travel time	37
Number of Players choosing Up	3
Number of Players choosing Down	5

**Table C4:** **Feedback example of *private payoff condition* and *private distribution condition***.

**Your decision**	**Down**
Your travel time	37

**Table C5:** **Preprogrammed agents in the *basic network phase***.

**Rounds**	**Down**	**Up**
Round 1	4	3
Round 2	4	3
Round 3	5	2
Round 4	3	4
Round 5	2	5
Round 6	4	3
Round 7	4	3
Round 8	3	4

**Table C6:** **Cooperative artificial population in the *augmented network phase***.

**Rounds**	**Down**	**UD**	**Up**
Round 9	2	0	5
Round 10	3	0	4
Round 11	3	0	4
Round 12	3	0	4
Round 13	5	0	2
Round 14	5	0	2
Round 15	4	0	3
Round 16	3	0	4

**Table C7:** **Competitive (Baseline condition) artificial population in the *aumented network phase***.

**Rounds**	**Down**	**UD**	**Up**
Round 9	2	3	2
Round 10	2	4	1
Round 11	0	5	2
Round 12	1	6	0
Round 13	1	6	0
Round 14	1	6	0
Round 15	1	6	0
Round 16	1	6	0

## References

[B1] Arnott R. Small K. (1994). The economics of traffic congestion. Am. Sci. 82, 446–455.

[B2] Asch S. E. (1951). Effects of group pressure upon the modification and distortion of judgments, in Groups, Leadership and Men; Research in Human Relations, ed Guetzkow H. (Oxford: Carnegie Press), 222–236.

[B3] Bardsley N. Sausgruber R. (2005). Conformity and reciprocity in public good provision. J. Econ. Psychol. 26, 664–681. 10.1016/j.joep.2005.02.001

[B4] Boyer R. Orléan A. (1997). Comment émerge la coopération? Recherches 2, 17–44.

[B5] Braess P. (1968). Über ein paradoxon aus der verkehrsplanung. Unternehmensforschung 12, 258–268.

[B6] Brewer M. B. Kramer R. M. (1986). Choice behavior in social dilemmas: effects of social identity, group size, and decision framing. J. Personal. Soc. Psychol. 50, 543–549. 10.1037/0022-3514.50.3.543

[B7] Dal Forno A. Merlone U. (2013). Replicating human interaction in Braess paradox, in Proceedings of the 2013 Winter Simulation Conference: Simulation: Making Decisions in a Complex World (Piscataway, NJ: IEEE Press), 1754–1765. 10.1109/wsc.2013.6721556

[B8] Dawes R. M. (1980). Social dilemmas. Annu. Rev. Psychol. 31, 169–193. 10.1146/annurev.ps.31.020180.001125

[B9] De Cremer D. Van Vugt M. (1999). Social identification effects in social dilemmas: a transformation of motives. Eur. J. Soc. Psychol. 29, 871–893. 10.1002/(SICI)1099-0992(199911)29:7<871::AID-EJSP962>3.0.CO;2-I

[B10] Faul F. Erdfelder E. Lang A.-G. Buchner A. (2007). G^*^ power 3: A flexible statistical power analysis program for the social, behavioral, and biomedical sciences. Behav. Res. Methods 39, 175–191. 10.3758/BF03193146 17695343

[B11] Fehr E. Schmidt K. M. (1999). A theory of fairness, competition, and cooperation. Q. J. Econ. 114, 817–868. 10.1162/003355399556151

[B12] Fischbacher U. (2007). z-Tree: Zurich toolbox for ready-made economic experiments. Exp. Econ. 10, 171–178. 10.1007/s10683-006-9159-4

[B13] Fisk C. Pallottino S. (1981). Empirical evidence for equilibrium paradoxes with implications for optimal planning strategies. Transport. Res. A Gen. 15, 245–248. 10.1016/0191-2607(81)90005-4

[B14] Gisches E. J. Rapoport A. (2012). Degrading network capacity may improve performance: private versus public monitoring in the braess paradox. Theory Decis. 73, 267–293. 10.1007/s11238-010-9237-0

[B15] Gneezy U. Rustichini A. (2000). Pay enough or don't pay at all. Q. J. Econ. 115, 791–810. 10.1162/00335530055491726604057

[B16] Irwin K. Edwards K. Tamburello J. A. (2015). Gender, trust and cooperation in environmental social dilemmas. Soc. Sci. Res. 50, 328–342. 10.1016/j.ssresearch.2014.09.002 25592940

[B17] Irwin K. Simpson B. (2013). Do descriptive norms solve social dilemmas? conformity and contributions in collective action groups. Soc. Forc. 91, 1057–1084. 10.1093/sf/sos196

[B18] Isaac S. Michael W. B. (1971). Handbook in Research and Evaluation. San Diego: ERIC.

[B19] Joireman J. A. Van Lange P. A. Kuhlman D. M. Van Vugt M. Shelley G. P. (1997). An interdependence analysis of commuting decisions. Eur. J. Soc. Psychol. 27, 441–463. 10.1002/(SICI)1099-0992(199707)27:4<441::AID-EJSP804>3.0.CO;2-S

[B20] Kelley H. H. Thibaut J. W. (1978). Interpersonal Relations: A Theory of Interdependence. New York, NY: Wiley.

[B21] Kolata G. (1990). What if they closed 42nd street and nobody noticed. N.Y. Times 25, 38.

[B22] Kollock P. (1998). Social dilemmas: The anatomy of cooperation. Ann. Rev. Sociol. 24, 183–214. 10.1146/annurev.soc.24.1.183

[B23] Komorita S. S. Parks C. D. (1996). Social Dilemmas. Boulder: Westview Press.

[B24] Liebrand W. B. (1984). The effect of social motives, communication and group size on behaviour in an n-person multi-stage mixed-motive game. Eur. J. Soc. Psychol. 14, 239–264. 10.1002/ejsp.2420140302

[B25] McClintock C. G. McNeel S. P. (1966). Reward and score feedback as determinants of cooperative and competitive game behavior. J. Personal. Soc. Psychol. 4, 606–613. 10.1037/h0023986

[B26] McNeel S. P. (1973). Training cooperation in the prisoner's dilemma. J. Exp. Soc. Psychol. 9, 335–348. 10.1016/0022-1031(73)90070-X

[B27] Molleman L. van den Berg P. Weissing F. J. (2014). Consistent individual differences in human social learning strategies. Nat. Commun. 5, 1–9. 10.1038/ncomms4570 24705692

[B28] Murchland J. D. (1970). Braess's paradox of traffic flow. Transport. Res. 4, 391–394. 10.1016/0041-1647(70)90196-6

[B29] Murphy R. O. Ackermann K. A. Handgraaf M. (2011). Measuring social value orientation. Judgm. Decis. Making 6, 771–781. 10.2139/ssrn.1804189

[B30] Ohtsuki H. Hauert C. Lieberman E. Nowak M. A. (2006). A simple rule for the evolution of cooperation on graphs and social networks. Nature 441, 502–505. 10.1038/nature04605 16724065 PMC2430087

[B31] Osborne M. J. Rubinstein A. (1994). A Course in Game Theory. Cambridge, MA: MIT Press.

[B32] Oskamp S. (1971). Effects of programmed strategies on cooperation in the prisoner's dilemma and other mixed-motive games. J. Confl. Resol. 15, 225–259. 10.1177/002200277101500207

[B33] Ostrom E. (1998). A behavioral approach to the rational choice theory of collective action: Presidential address, american political science association, 1997. Am. Polit. Sci. Rev. 92, 1–22. 10.2307/2585925

[B34] Pala M. G. Baltazar S. Liu P. Sellier H. Hackens B. Martins F.. (2012). Transport inefficiency in branched-out mesoscopic networks: an analog of the Braess paradox. Phys. Rev. Lett. 108:076802. 10.1103/PhysRevLett.108.076802 22401236

[B35] Parks C. D. Xu X. Van Lange P. A. (2015). Does information about others' behavior undermine cooperation in social dilemmas? Group Process. Intergroup Relat. 10.1177/1368430215612220. [Epub ahead of print].

[B36] Patchen M. (1987). Strategies for eliciting cooperation from an adversary laboratory and internation findings. J. Confl. Resol. 31, 164–185. 10.1177/0022002787031001009

[B37] Perc M. Szolnoki A. (2010). Coevolutionary games: a mini review. BioSystems 99, 109–125. 10.1016/j.biosystems.2009.10.003 19837129

[B38] Rapoport A. Kugler T. Dugar S. Gisches E. J. (2009). Choice of routes in congested traffic networks: experimental tests of the braess paradox. Games Econ. Behav. 65, 538–571. 10.1016/j.geb.2008.02.007

[B39] Rapoport A. Mak V. Zwick R. (2006). Navigating congested networks with variable demand: experimental evidence. J. Econ. Psychol. 27, 648–666. 10.1016/j.joep.2006.06.001

[B40] Roughgarden T. (2006). On the severity of braess's paradox: designing networks for selfish users is hard. J. Comput. Syst. Sci. 72, 922–953. 10.1016/j.jcss.2005.05.009

[B41] Samuelson C. D. Messick D. M. Rutte C. Wilke H. (1984). Individual and structural solutions to resource dilemmas in two cultures. J. Personal. Soc. Psychol. 47, 94–104. 10.1037/0022-3514.47.1.94

[B42] Skinner B. (2010). The price of anarchy in basketball. J. Quantit. Analy. Sports 6, 1–9. 10.2202/1559-0410.1217

[B43] Smith V. L. (1991). Rational choice: the contrast between economics and psychology. J. Polit. Econ. 99, 877–897. 10.1086/261782

[B44] Steinberg R. Zangwill W. I. (1983). The prevalence of Braess paradox. Transport. Sci. 17, 301–318. 10.1287/trsc.17.3.301

[B45] Stouten J. De Cremer D. Van Dijk E. (2005). All is well that ends well, at least for proselfs: emotional reactions to equality violation as a function of social value orientation. Eur. J. Soc. Psychol. 35, 767–783. 10.1002/ejsp.276

[B46] Stouten J. De Cremer D. Van Dijk E. (2009). When being disadvantaged grows into vengeance: the effects of asymmetry of interest and social rejection in social dilemmas. Eur. J. Soc. Psychol. 39, 526–539. 10.1002/ejsp.556

[B47] Szolnoki A. Perc M. (2015). Conformity enhances network reciprocity in evolutionary social dilemmas. J. R. Soc. Interf. 12:20141299. 10.1098/rsif.2014.1299 25540242 PMC4305429

[B48] Treece E. W. Treece J. W. Jr (1977). Elements of research in nursing. Nurs. Res. 26, 239 10.1097/00006199-197705000-00032

[B49] Van Lange P. A. (1999). The pursuit of joint outcomes and equality in outcomes: an integrative model of social value orientation. J. Personal. Soc. Psychol. 77, 337–349. 10.1037/0022-3514.77.2.337

[B50] Van Lange P. A. Joireman J. Parks C. D. Van Dijk E. (2013). The psychology of social dilemmas: a review. Organ. Behav. Hum. Decis. Process. 120, 125–141. 10.1016/j.obhdp.2012.11.003

[B51] Van Lange P. A. M. Rusbult C. E. (2011). Interdependence theory, in Handbook of Theories of Social Psychology, eds Van Lange P. A. M. Kruglanski A. W. Higgins E. T. (Thousand Oaks, CA: Sage), 251–272.

[B52] Wade-Benzoni K. A. Tenbrunsel A. E. Bazerman M. H. (1996). Egocentric interpretations of fairness in asymmetric, environmental social dilemmas: explaining harvesting behavior and the role of communication. Organ. Behav. Hum. Decis. Process. 67, 111–126. 10.1006/obhd.1996.0068

[B53] Wang Z. Wang L. Szolnoki A. Perc M. (2015). Evolutionary games on multilayer networks: a colloquium. Eur. Phys. J. B 88, 1–15. 10.1140/epjb/e2015-60270-7

[B54] Wilson W. (1971). Reciprocation and other techniques for inducing cooperation in the prisoner's dilemma game. J. Confl. Resol. 15, 167–195. 10.1177/002200277101500205

[B55] Wu J. Balliet D. Van Lange P. A. M. (2015). When does gossip promote generosity? indirect reciprocity under the shadow of the future. Soc. Psychol. Personal. Sci. 6, 923–930. 10.1177/1948550615595272

[B56] Xiao E. Kunreuther H. (2015). Punishment and cooperation in stochastic social dilemmas. J. Confl. Resol. 59, 1–24. 10.1177/0022002714564426

[B57] Youn H. Gastner M. T. Jeong H. (2008). Price of anarchy in transportation networks: efficiency and optimality control. Phys. Rev. Lett. 101, 1–4. 10.1103/PhysRevLett.101.128701 18851419

